# Study on characteristics of soil and nutrient losses in Sunjiagou small watershed in cold black soil area

**DOI:** 10.1371/journal.pone.0289479

**Published:** 2023-08-03

**Authors:** Taoyan Dai, Liquan Wang, Tienan Li, Pengpeng Qiu, Jun Wang, Zhengjun Wang

**Affiliations:** 1 College of Water Conservancy and Electric Power, Heilongjiang University, Harbin, China; 2 Heilongjiang Province Hydraulic Research Institute, Harbin, China; Jinan University, CHINA

## Abstract

Investigating the impact of different factors on soil and nutrient loss and suggesting viable control measures is currently a significant concern. This study aims to examine the variations in soil erosion, as well as nitrogen and phosphorus loss, in the core area of the typical hilly diffuse Blackland erosion control. To achieve this, runoff plots with slopes of 3° and 5° were set up in the Sunjiagou sub-basin, located in the upper reaches of the Feiketu River. These plots were subjected to various soil and water conservation measures, along with different levels of vegetation cover. This study aims to analyze the soil and nutrient loss patterns and characteristics in each runoff plot during the natural rainfall events occurring between 2020 and 2022. The results show that soil and nutrient losses are highly significantly and positively correlated with rainfall intensity. The RUSLE model demonstrates a better fit for both cross ridge tillage and bare ground. The loss of nitrogen was much more significant than that of phosphorus, and nitrate nitrogen is the main form of nitrogen loss. Nitrogen loss is mainly dominated by nitrate nitrogen (NN), which is easily soluble in water and constantly migrates with runoff due to the negatively charged NN (NN accounted for 45.2% ~ 81.8% of total nitrogen (TN)). In contrast, the positively charged ammonia nitrogen (AN) is more stable in combination with the soil; large losses only occur under severe sediment erosion. Phosphorus is easily attached to sediment, and the high sediment production leads to a more serious loss of total phosphorus (PP) in the particulate state (PP accounts for 72.7% ~ 96.2% of total phosphorus (TP)). Changing longitudinal ridge tillage to cross ridge tillage and planting vegetation with better water retention and sediment fixation as plant hedges can effectively prevent the loss of soil, runoff, nitrogen, and phosphorus.

## Introduction

Non-point source pollution has become a significant source of water pollution and is more difficult to prevent. Nitrogen and phosphorus loss caused by soil erosion reduces soil productivity [1] and leads to eutrophication of water bodies [2, 3]. Non-point source pollution is a global problem and has been the subject of much research in countries worldwide [4–8]. In China, agriculture has surpassed industry as the primary source of non-point source pollution. Erosion and sediment-related chemical transport should be given more attention in rural China [9]. Due to soil erosion from cultivated land, nitrogen and phosphorus are exported in the dissolved runoff or the particulate state of sediments, thus polluting water bodies.

As one of the four major black soil regions globally, the Northeast Black Soil Region is the stabilizer of China’s food market. Black soil has also been compared to "the giant panda of cultivated land". However, with the interaction of natural and human factors in the black soil area, soil erosion has become more and more serious, leading to the thinning of the black soil layer and the decrease of soil fertility [10]; coupled with a large amount of chemical fertilizer input and the scientific arable land management system is not perfect, leading to nutrient loss and water pollution [11–13]. At present, the surface soil of sloping arable land in black soil areas is lost at a rate of 2~3 mm·a^-1^, and the organic matter content of the surface soil decreases at a rate of 5‰ per year.

A number of studies have been conducted on the issue of nonpoint source pollution. Zhu et al. [14] analyzed the spatial and temporal evolution of the output risk probability of agricultural nonpoint source pollution in the Fuling district using the SWAT model and output risk model. Huang et al. [15] identified the critical source areas of nonpoint source pollution more accurately through a rainfall-driven correlation-based mapping method (PCM). However, such studies have been carried out within the catchment scale [6, 8, 16–18], Only a few studies on soil and nutrient loss characteristics at the plot scale [19]. Soil erosion and nutrient loss studies using runoff plots have been more effectively utilized in China, the United States and Europe [20–22]. Runoff plots were used to explore the effects of no-till practices on reducing agricultural nonpoint source pollution [23] and the effects of variable versus uniform fertilizer application on nutrient loss [24]. The variables in runoff plots are easier to control than in small watersheds. Setting up runoff plots in small watersheds ensures that climate, soil, and other factors are the same. The experiments in runoff plots have led to the scientific and rational use of engineering and biological measures to control nutrient loss, which provides a basis for reducing nonpoint source pollution in small watersheds.

Nowadays, the problem of soil erosion is studied mainly using artificial rainfall and remote sensing image analysis [25–28]. Wang et al. [29] analyzed the soil erosion characteristics of the black soil area in the middle and lower reaches of the Mu Shi River based on GIS and RS technology. Still, this type of study lacks calibration and verification of the results and analysis due to the lack of ground monitoring in the study area. Li et al. [30] analyzed the soil erosion in the red soil area of southern China by simulating rainfall. Although the erosion mechanism can be understood more clearly, it is different from the natural rain. This leads to differences with the erosion characteristics under natural rainfall.

The study area is a typical hilly, where the process of nonpoint source pollution is complex and difficult to prevent. However, most studies on nonpoint source pollution have been conducted in red soil areas, loess areas and purple soil areas, and the analysis of nitrogen and phosphorus losses and their influencing factors in black soil areas has rarely been covered. The objectives of this study are (i) to characterize the variations in runoff, soil erosion, and the loss of each form of nitrogen and phosphorus; (ii) exploring the fitting effect of RUSLE on soil erosion under different conditions; and (iii) to study the response of soil and nutrient loss to soil and water conservation measures. In the end, some suggestions are made to manage soil and nutrient loss in black soil areas.

## Materials and methods

### Study area

**Fig 1:**
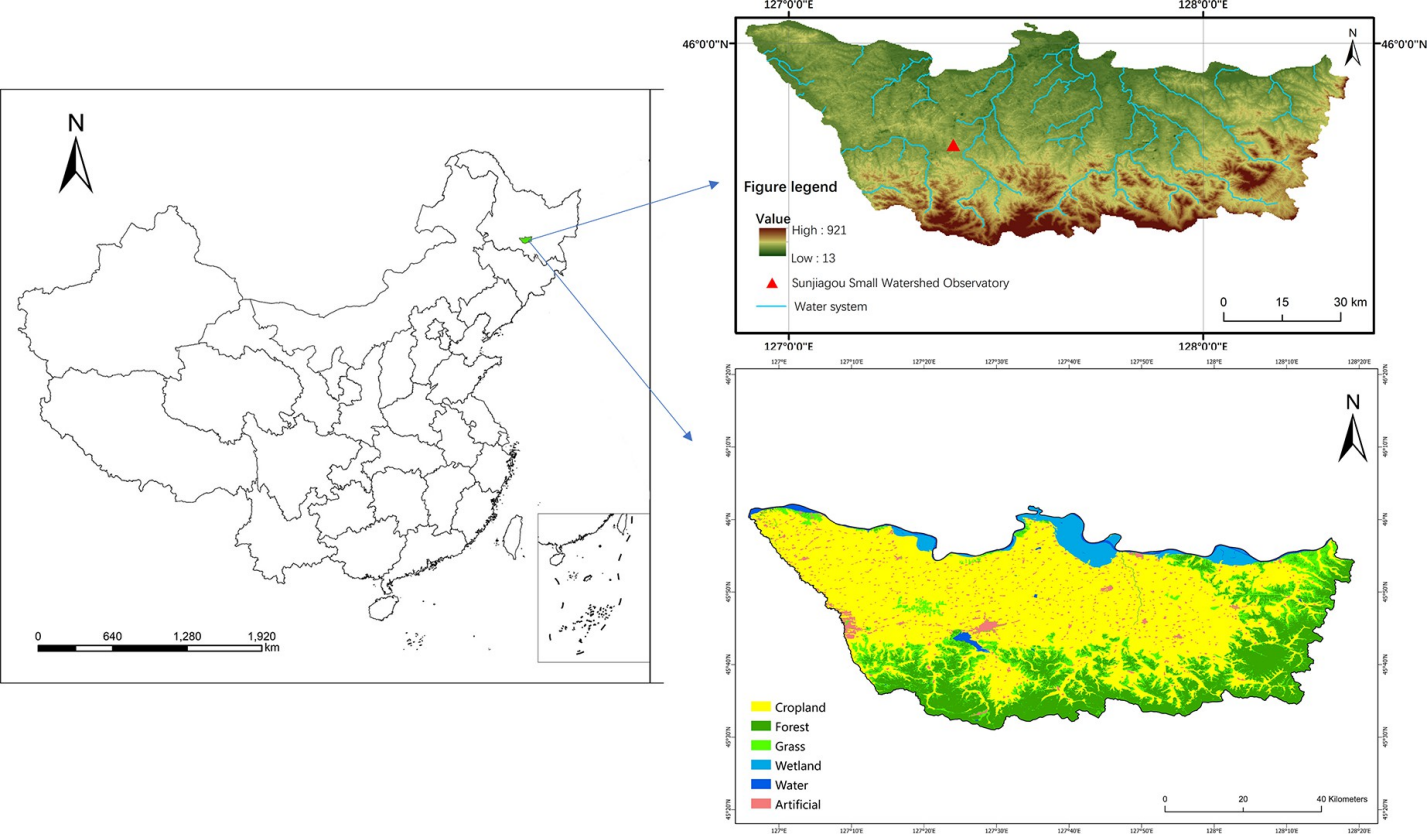
Location of the study area.

The Sunjiagou small watershed is located in Bin County, Harbin City, Heilongjiang Province. The runoff field of Bin County Water Conservation Science and Technology Park is the ground monitoring station of the Sunjiagou small watershed, located at 127°24′47″E and 45°44′57″N. Bin County Water Conservation Science and Technology Park is also a national soil and water conservation monitoring station (Fig 1). The watershed is located in the mid-temperate continental monsoon climate zone, with a multi-year average rainfall of 681mm and an average annual temperature of 4.1°C. Rainfall is unevenly distributed within the year, mainly concentrated in June-September, with precipitation in July and August in summer accounting for about 60% of the year. A total of 91 rainfall events were monitored from 2020 to 2022, and the number of rainfall events that resulted in runoff generation was 31, with a total annual rainfall of 600.6 mm and a rainfall erosion force of 2900.86 MJ·mm·(hm^2^·h)^-1^. Soil types are mainly black soils, but also include white pulpy soils, meadow soils, and dark brown soils. Soil thickness was 20 cm and bulk weight of 1.33~1.35g-cm^-3^. The content of micro agglomerates (agglomerated structural units 0.05–0.25 mm in diameter) ranged from 73.23% to 78.52% and 61.68% to 70.84% in bare land and cultivated land, respectively. Mainly planted with *Zea mays*, *Oryza sativa L*, and *Glycine max*, and soil erosion occurs primarily on sloping land and wasteland.

### Runoff plots

**Fig 2:**
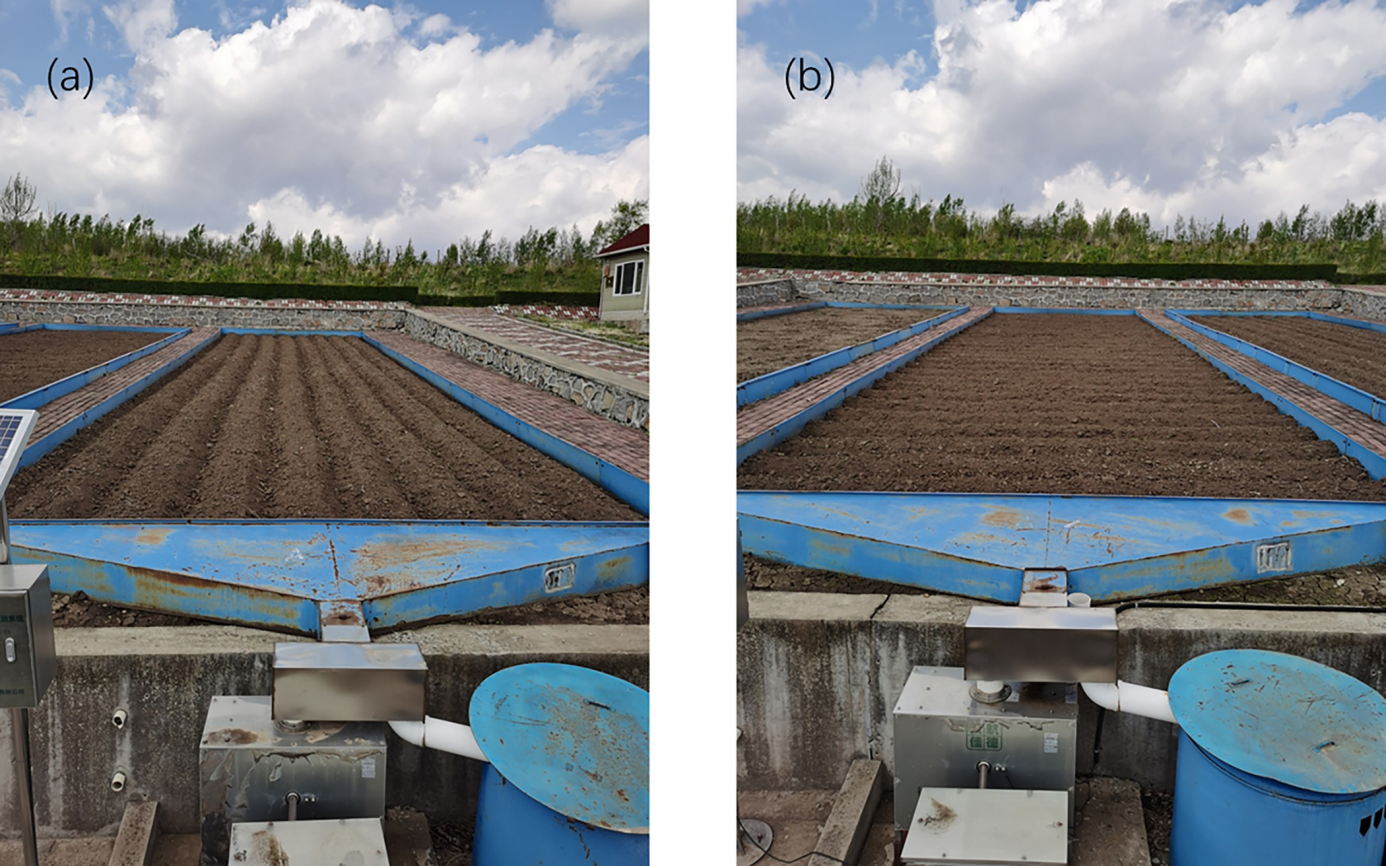
Two types of tillage: (a) longitudinal ridge tillage, (b) cross ridge tillage.

Six runoff plots in the runoff field were selected as research objects, and the specific layout is shown in Table 1. The form requires that their slope, soil and water conservation measures, vegetation type, and other factors represent the local area, and all farming activities are carried out according to the farming habits of local farmers. For each runoff plot, 15 kg of compound fertilizer was applied as a base fertilizer before planting. The ratio of N, P_2_O_5_, and K_2_O in the compound fertilizer is 15:35:10. The seeds were sown on May 18, at a depth of 10 cm, and spray pesticides on May 11, June 11, July 16 and August 27. The size of the runoff plots was 5 m × 20 m; the thickness of the black soil layer was about 20 cm. Most of the sloping land in northeast China is dominated by gentle slopes; therefore, 3° and 5° runoff plots were used in this study. *Amorpha fruticosa Linn*. was used as a plant hedge in this study because it is a common plant in northeastern China and has good water retention and sediment fixation effects. And *Glycine max*, a major crop in the Northeast, was selected for planting in the runoff plots. Soil and water and nutrient loss were analyzed from various aspects such as slope, soil and water conservation measures (Fig 2), and vegetation cover.

**Table 1:** Information of the study runoff plots.

Plot	Land Use	Slope (°)	Soil conservation measure	Vegetation
A_1_	Cultivated	3	longitudinal ridge tillage (distance:70cm)	*Glycine max (Linn*.*) Merr*. (30 plants per row)
A_2_	Cultivated	3	cross ridge tillage (distance:70cm)	*Glycine max (Linn*.*) Merr*. (30 plants per row)
A_3_	Bare land	3	-	-
B_1_	Cultivated	5	longitudinal ridge tillage (distance:70cm)	*Glycine max (Linn*.*) Merr*. (30 plants per row)
B_2_	Cultivated	5	cross ridge tillage and plant hedge (distance:70cm)	*Glycine max (Linn*.*) Merr*. (30 plants per row) and *Amorpha fruticosa Linn*.
B_3_	Bare land	5	-	-

### Data collection

The runoff volume is measured by the volume of water in the diversion bucket and the runoff bucket, and the sediment production is measured by the total amount of sediment in the diversion bucket as well as in the catch basin. The precipitation observation equipment uses fully automatic weather stations and siphon type self-registering rain gauges to monitor rainfall, I_30_, and rainfall erosion force indicators. Instantaneous water samples were collected at 0.5h intervals during the first two hours of rainfall and then at one-hour intervals until the end of the rain, collected in 500ml plastic bottles, stored in a 4°C refrigerator, and returned to the laboratory in time for timely testing. Test items include total phosphorus (TP), total nitrogen (TN), total soluble phosphorus (DTP), total particulate phosphorus (PP), total soluble nitrogen (DTN), ammonia nitrogen (AN), nitrate-nitrogen (NN). The TN and TP indicators were measured by shaking the water sample and taking a small amount of liquid directly, while the AN, NN, DTN and DTP indicators needed to be measured by filtering the water sample with 0.45 μm filter membrane. The specific operations were referred to the corresponding national standards (Table 2) and finally measured using a UV-Vis spectrophotometer (Evolution 220; Thermo Fisher, USA).

**Table 2:** Water quality test indexes and methods.

Test index	Test Method
Sediment	Weighing method
NN	Phenol disulfonic acid spectrophotometric method (HJ/T3462007)
AN	Nascent reagent spectrophotometry (HJ535-2009)
TP,DTP	Potassium persulfate oxidation—molybdenum antimony anti-colorimetric method (GB/T11893-1989)
TN, DTN	Oxidation of basic potassium persulfate—UV spectrophotometric method (GB/T11894-1989)

### Data processing

Event mean concentration (EMC) is used to calculate the average concentration of each rainfall, and EMC (mg·L^-1^) is the weighted average concentration of runoff pollutants, which can be expressed as:

$$EMC=\frac{\sum_{i=1}^{n-1}\frac{Q_{i}c_{i}+Q_{i+1}c_{i+1}}{2}\times \Delta t}{\sum_{i=1}^{n-1}\frac{Q_{i}+Q_{i+1}}{2}\times \Delta t}$$ (1)

Where n is the number of samples; △t is the time interval between two adjacent samples (s); c_i_ and c_i+1_ are the concentration of this nutrient at stage i and i+1 sampling (mg·L^-1^); Q_i_ and Q_i+1_ are the runoff rates at the adjacent monitoring moments (m^3^·s^-1^).

In this paper, the erosion of rainfall is estimated by modifying the universal soil loss equation (RUSLE) with the following equation:

A=R×K×LS×C×P (2)

A is the soil erosion modulus (t km^-2^); R is the rainfall erosion force factor (MJ mm (hm^2^ h)^-1^); K is the soil erodibility factor ((t hm^2^ h)/(hm^2^ MJ mm)); L is the slope length factor; S is the slope factor; C is the vegetation cover and crop management factor; and P is the soil and water conservation factor.

Referring to the method of Zhang et al. [31] to calculate rainfall erosion forces by rainfall volume and intensity:

$$EI_{30}=0.1773(PI_{10})$$ (3)

P is the rainfall amount, I_10_ is the maximum 10 min rain intensity, and PI_10_ unit is (mm^2^ h^-1^). The slope length factor L was calculated by referring to the study of Fu et al. [32]. The slope length factor S was calculated using the method of McCool et al. [33] in the area below 10° and Liu et al. [34] in the area above 10°. The equations are as follows:

$$L={\left(\frac{\lambda }{22.13}\right)}^{m}$$ (4)

$$S=\{\begin{array}{l} 10.80\sin\theta +0.03\;\theta <5{}^{\circ}\\ 16.80\sin\theta -0.5\;5{}^{\circ}\leq \theta <10{}^{\circ}\\ 21.91\sin\theta -0.96\;10{}^{\circ}\leq \theta \end{array}$$ (5)

λ is the slope length; m is the slope length index; and θ is the slope degree (°). Other factors were calculated with reference to the study of Zhang et al. [35] in Heilongjiang Province. The vegetation cover and crop management factor C for soybean was determined based on the weighted average of C values for each growth period of soybean. the C value for runoff plots with soybean cover was 0.2626 and for bare ground was 1. the P value for longitudinal ridge tillage was 1 and for cross ridge tillage was 0.352. the K value for black soil was taken as 0.26.

The experimental data of this study were counted and calculated using Excel 2016, and Pearson correlation analysis was performed using SPSS 25.0 and plotted with OriginPro 2021.

## Results and analysis

### Surface runoff and soil loss

**Fig 3:**
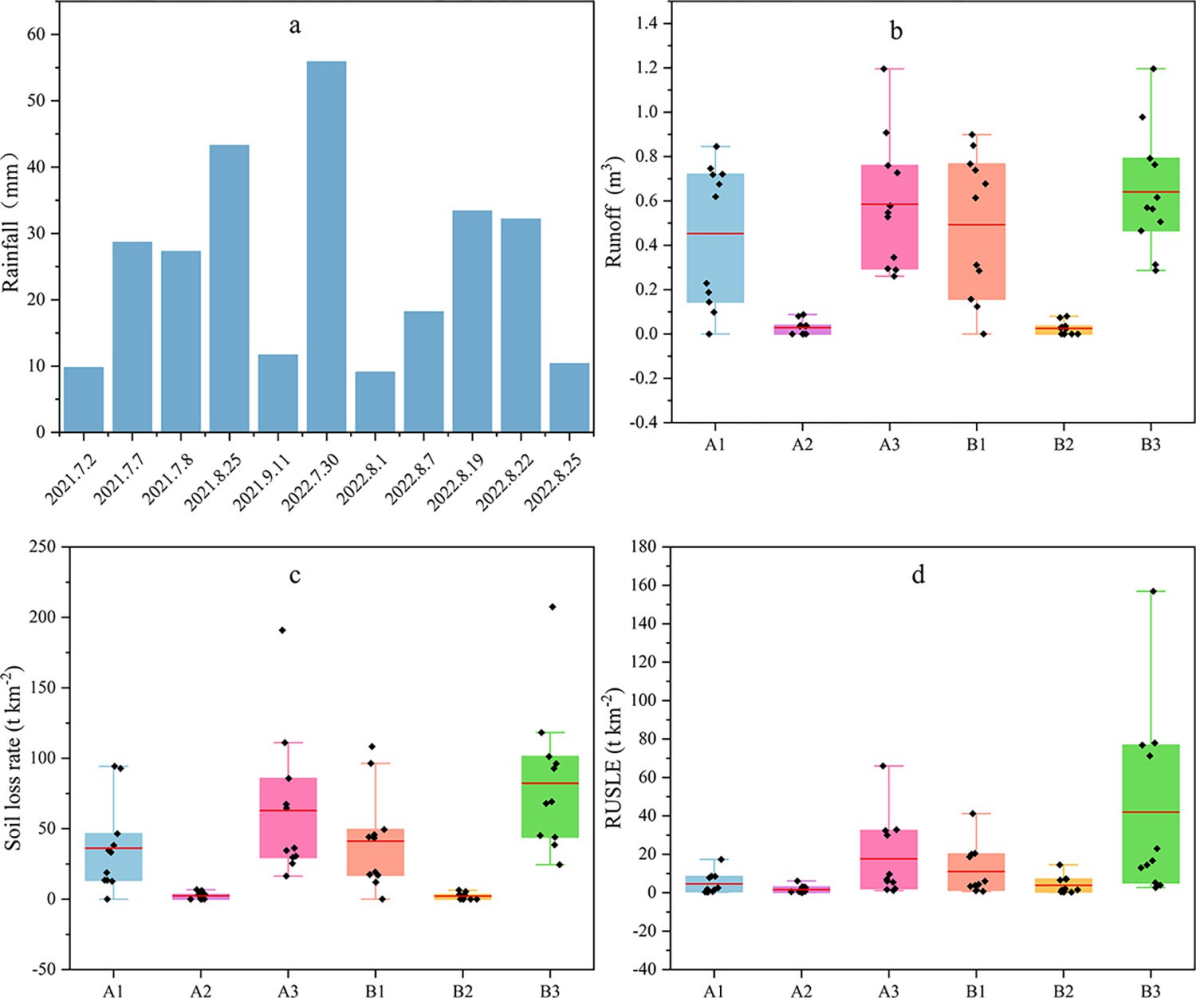
Rainfall (a), runoff volume (b), soil erosion rate (c) and RUSLE predictions (d) for each runoff plot.

The trends of runoff production, sediment production, and rainfall in each plot were similar (Fig 3). Under the typical rainfall of each field, the order of flow production, as well as the size of sediment production in each runoff plot, is basically: 5° cross-slope furrow tillage with a planted hedge (plot B_2_) < 3° cross-slope furrow tillage (plot A_2_) < 3° down-slope furrow tillage (plot A_1_) < 5° down-slope furrow tillage (plot B_1_) < 3° bare land (plot A_3_) < 5° bare land (plot B_3_).

### Nitrogen and phosphorus loss

**Fig 4:**
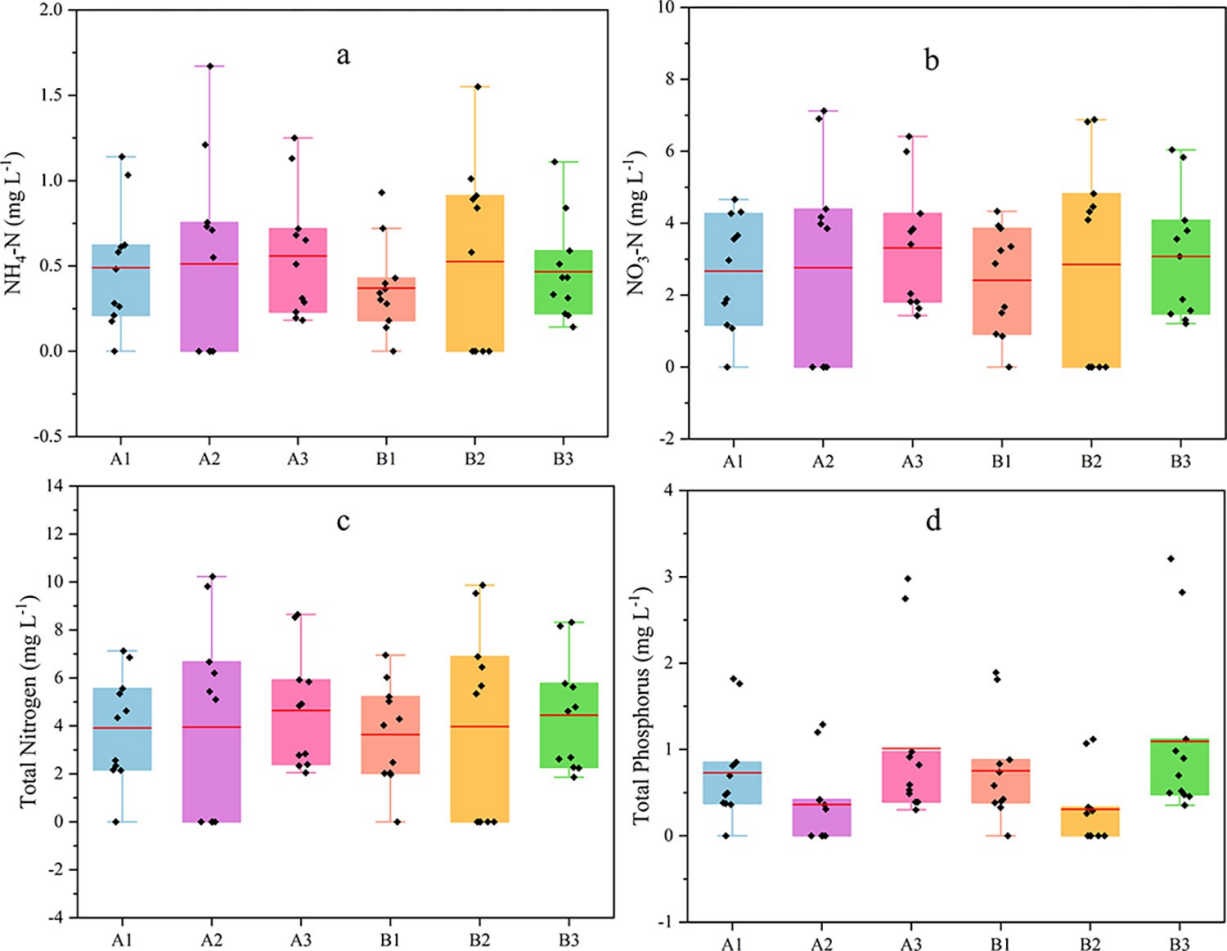
Loss concentrations of ammonia nitrogen, nitrate nitrogen, total nitrogen and total phosphorus in each runoff plot.

Event mean concentration (EMC) was used to calculate the average concentration of AN, NN, TN, and TP in each rainfall event and the amount lost in a single rainfall event (Fig 4).

AN, NN, and TN have relatively similar loss characteristics and fit well with rainfall (Fig 4). The nitrogen of each form varied widely: 0.14 mg·L^-1^ ~ 1.67 mg·L^-1^ for AN, 0.86 mg·L^-1^ ~ 7.12 mg·L^-1^ for NN, and 1.86 mg·L^-1^ ~ 10.23 mg·L^-1^ for TN. Loss fluxes of each form of nitrogen: 0.26 kg km^-2^ ~13.51 kg km^-2^ for AN, 1.06 kg km^-2^ ~71.61 kg km^-2^ for NN, and 2.12 kg km^-2^ ~101.98 kg km^-2^ for TN.

**Fig 5:**
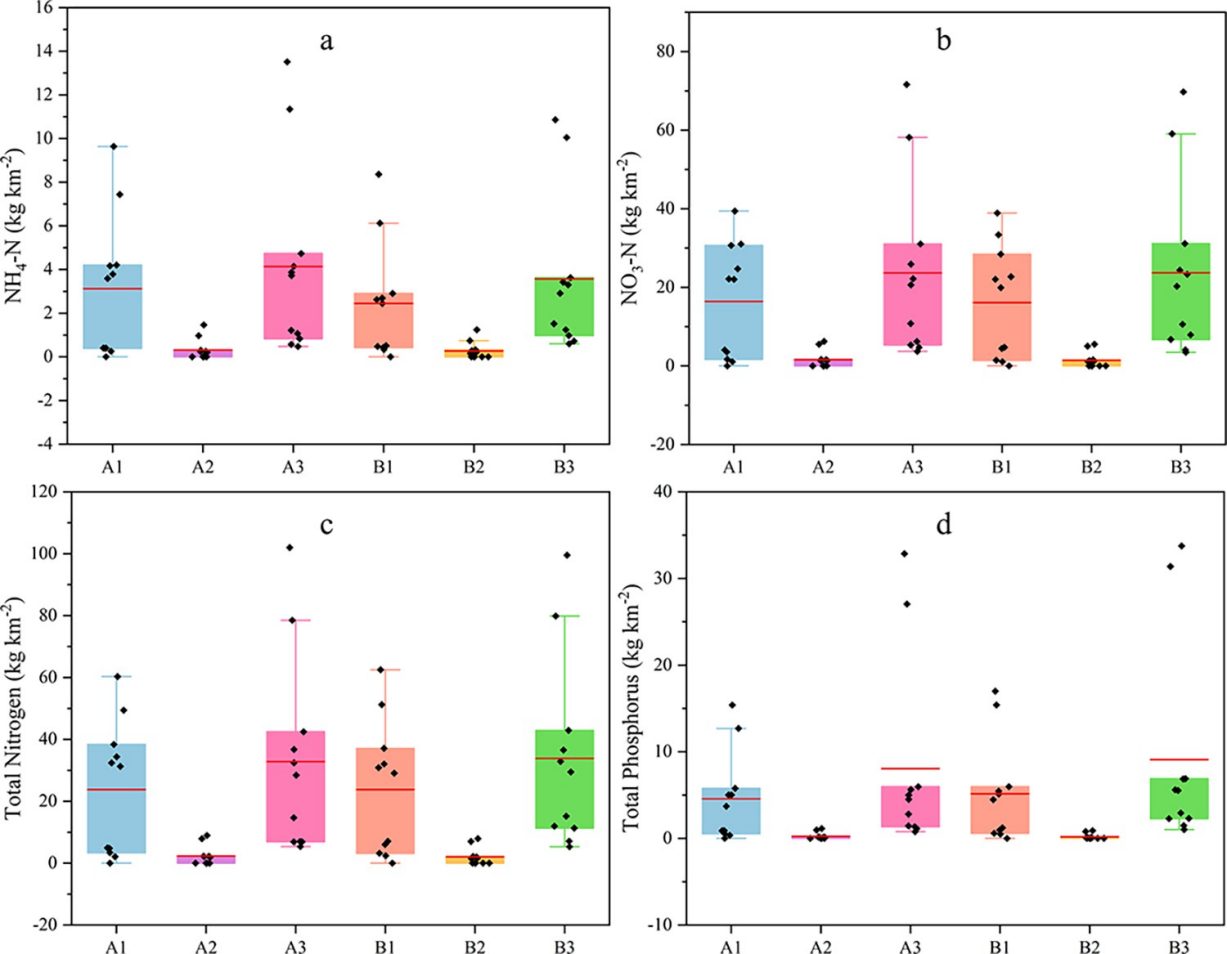
Loss fluxes of ammonia nitrogen, nitrate nitrogen, total nitrogen and total phosphorus in each runoff plot.

The trend of phosphorus loss is similar to that of rainfall, and the trend of TP is relatively stable due to the strong fixation capacity of phosphorus (Fig 5). The loss concentration of TP ranges from 0.30 mg·L^-1^ ~ 2.98 mg·L^-1^ and the flux of TP ranges from 0.36 kg km^-2^ ~ 32.85 kg km^-2^.

**Fig 6:**
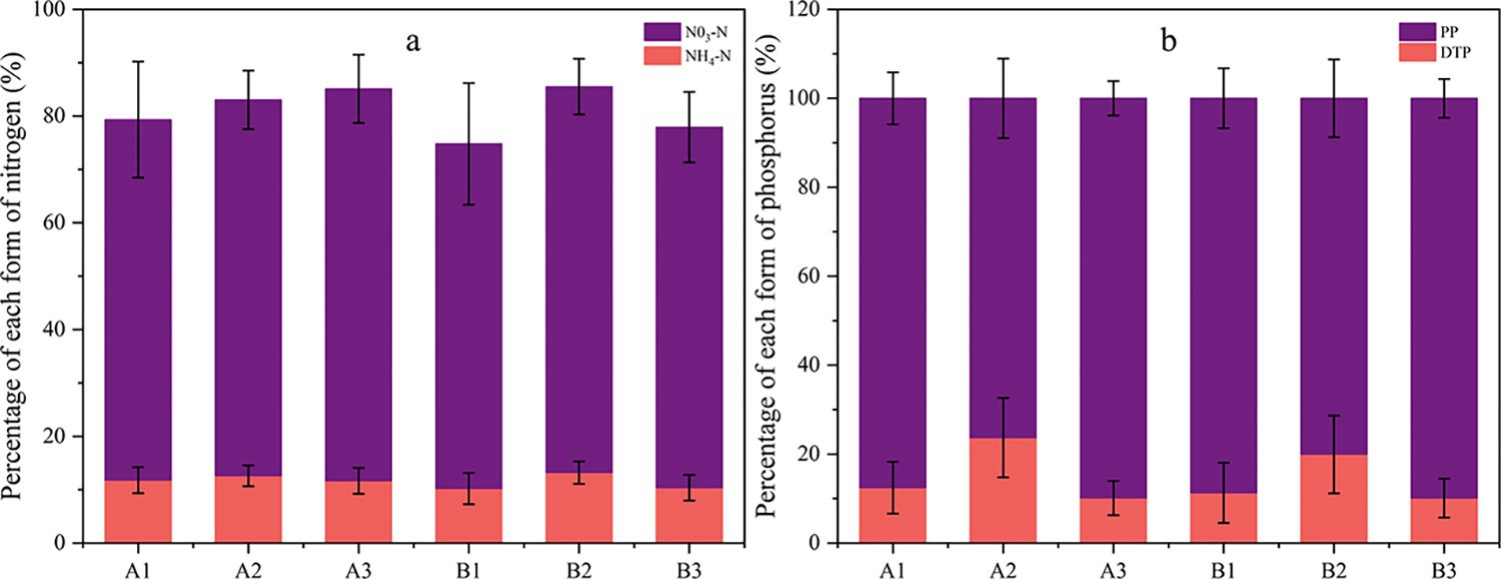
Percentage of each form of nitrogen (a) and phosphorus (b).

Under different soil and water conservation measures, the loss of DTN accounts for 60.1% ~ 92.6% of TN; the loss of NN accounts for 45.2% ~ 81.8% of TN; and the loss of AN accounts for 6.3% ~ 16.1% of TN (Fig 6A). The variability of PP and DTP was small in five typical rainfall events (Fig 6B). The loss flux of PP accounted for 72.7% ~ 96.0% of TP.

### Correlation analysis of nutrient loss and soil erosion

Pearson correlation analysis was performed on each form’s rainfall, runoff, sediment production, and nitrogen and phosphorus loss (Table 3). The correlation coefficients between rainfall and nitrogen and phosphorus loss were greater than 0.9. The relationship between AN, NN and TN and runoff is more obvious.

**Table 3:** Pearson correlation coefficients between each form of nitrogen and phosphorus and rainfall, runoff, and soil erosion rate (SLR) in six runoff plots.

Runoff Plot	Relevance	AN	NN	TN	PP	TP
	Rainfall	0.982**	0.946**	0.971**	0.956**	0.964**
A_1_	Runoff	0.887**	0.970**	0.954**	0.790**	0.806**
	SLR	0.968**	0.874**	0.918**	0.981**	0.987**
	Rainfall	0.919**	0.930**	0.932**	0.863**	0.877**
A_2_	Runoff	0.936**	0.968**	0.964**	0.904**	0.916**
	SLR	0.898**	0.951**	0.948**	0.877**	0.891**
	Rainfall	0.904**	0.902**	0.896**	0.833**	0.839**
A_3_	Runoff	0.938**	0.964**	0.966**	0.879**	0.881**
	SLR	0.885**	0.865**	0.870**	0.901**	0.906**
	Rainfall	0.965**	0.957**	0.978**	0.943**	0.950**
B_1_	Runoff	0.850**	0.970**	0.949**	0.815**	0.831**
	SLR	0.958**	0.921**	0.946**	0.969**	0.980**
	Rainfall	0.934**	0.929**	0.929**	0.857**	0.858**
B_2_	Runoff	0.951**	0.973**	0.969**	0.915**	0.918**
	SLR	0.869**	0.936**	0.929**	0.849**	0.862**
	Rainfall	0.921**	0.909**	0.899**	0.845**	0.859**
B_3_	Runoff	0.902**	0.959**	0.962**	0.891**	0.886**
	SLR	0.860**	0.926**	0.932**	0.869**	0.860**

注: "**" and"*" represent significant at p = 0.01 and 0.05, respectively.

## Discussion

### Analysis of the factors influencing soil erosion

Rainfall and runoff are the driving force of soil erosion, and rainfall amount, duration, and intensity affect the flow and sediment production of each runoff plot [36]. The short duration and high volume of rainfall produced changes in soil structure that led to more severe erosion [37]. Continuous rainfall makes the soil wetter, resulting in increased soil water content and more likely to produce runoff, thus causing more severe erosion [38].

Plant cover also significantly affected soil erosion, especially under heavy rainfall. Some scholars have suggested that longitudinal ridge tillage will increase soil erosion, which is different from the present study. In the present study, A_1_ and B_1_ plots with longitudinal ridge tillage had a certain effect on water retention and sediment fixation compared with bare land, mainly because the vegetation cover of A_1_ and B_1_ plots played a key role. Due to the rapid growth of *Glycine max* in the rainy season, the vegetation cover ranged from 20%-35% in late June to 70%-80% in mid-to early July, and even up to 90% in August. The high vegetation cover dramatically reduces soil erosion [39]. The stems and leaves of the vegetation had an enhanced ability to intercept rainfall, reducing the kinetic energy of raindrops and reducing the direct splash of raindrops on the soil [40]; the surface apoplast protects the soil surface from direct raindrop impact and significantly enhances infiltration [41]. The plant roots were continuously developed, improving the physical and chemical properties of the soil, enhancing the water-holding capacity, and enriching the structure of the aggregates, which led to an enhanced water retention function of the vegetation [42, 43]. It can be seen that the former dominates the effect of vegetation cover and whether or not longitudinal ridge tillage on soil erosion in this study area under the weather of heavy rainfall.

The flow and sediment production in plot A_2_ and plot B_2_ are relatively flat compared with those of other runoff cells and are much lower than those of other runoff cells. This indicates that the flow and sediment production in A_2_ and B_2_ plots are much lower than those in other runoff plots, which play a perfect role in water retention and sediment fixation and significantly reduce the soil erosion in black land. In multiple rainfall events, plot A2 and plot B2 did not produce runoff, indicating that the cross ridge tillage measures were more prominent in the light rainfall weather. In plot A_2_, compared with plot A_1_, the cross ridge tillage reduced runoff losses by 88.8% to 95.0% and sediment losses by 90.2% to 93.5% compared with the longitudinal ridge tillage. Compared with the longitudinal ridge tillage and cross ridge tillage, the ridges of the cross ridge tillage intercept runoff and increases the contact time between runoff and soil, which makes most of the runoff infiltrate into the ground [44]. Unlike plot A_2_, plot B_2_ was planted with *Amorpha fruticosa Linn*. as a plant hedge based on cross ridge tillage. In the horizontal comparison of plot B_1_, the runoff loss was reduced by 91.3%~96.7%, and the sediment loss was reduced by 92.7%~94.5%; in terms of annual rainfall, the runoff loss was reduced by 96.5%, and the sediment loss was reduced by 97.5%. Planting purple acacia as a plant hedge is more effective in reducing soil erosion; purple acacia is a typical soil and water conservation crop; its root system is conducive to improving soil structure to play a better role in water retention and sediment fixation [45]. Yuan et al. [46] concluded that cross ridge tillage could reduce sediment loss by 43.43%~93.9% compared with longitudinal ridge tillage; the water retention and sediment fixation capacity of the cross ridge tillage was not as outstanding as in the present study. This is due to the low rainfall and gentle slope in this study area, where the monopoles can intercept most of the loss without causing the ridges to break [38]. Numerous scholars [47–49] have obtained similar results and values in different experimental studies and concluded that cross ridge tillage and increased plant hedges could significantly reduce soil erosion.

The RUSLE model is commonly used for soil erosion estimation. Therefore, it is important to investigate whether the RUSLE model fits accurately under different land use types, soil and water conservation measures, and other factors. The estimated values of RUSLE for cross ridge tillage (plots A2, B2) and bare land plots (A3, B3) are closer to the measured values and can be used for estimating soil loss in this terrain in the watershed (Fig 3C and 3D). However, the difference between the estimated value and the measured value of the longitudinal ridge tillage (A1 and B1 plots) is relatively large. The reason may be that the value of soil and water conservation factor P is small under the condition of longitudinal ridge tillage. Future studies should focus on fitting RUSLE in the longitudinal ridge tillage plots and explore the value of soil and water conservation factors.

### Analysis of factors influencing nitrogen and phosphorus loss

Different soil and water conservation measures also affected nutrient loss. Excluding the two rainfall events in which the cross ridge tillage did not produce flow, the concentration of AN, NN, and TN loss in plot A_1_ was much greater than that in the cross ridge tillage land and bare ground (Fig 4). Compared with longitudinal ridge tillage and bare ground, the monopod of cross ridge tillage intercepts runoff. It prolongs the contact time between runoff and topsoil, while nitrogen is easily dissolved in water and primarily lost in dissolved form. The amount of sediment produced by cross ridge tillage land is much smaller than that of longitudinal ridge tillage land and bare land, even though the loss concentration is higher; in contrast, the amount of loss of cross ridge tillage land is much smaller than that of longitudinal ridge tillage land and bare land. Compared with the longitudinal ridge tillage plot A_1_, the loss of AN, NN, and TN in the cross ridge tillage plot A_2_ was reduced by 84.9%~94.3%, 82.2%~94.9%, and 84.1%~94.2%, respectively. Fang [50] showed that contour tillage could significantly reduce TN and TP losses and indicated that cross ridge tillage is desirable for gentle slopes. Xi et al. [51] showed that cross ridge tillage cut nitrogen loss by 47.09% to 78.45% compared to longitudinal ridge tillage. Compared to this study, the proportion of nitrogen reduction in this paper was greater due to the simulated rainfall of 60 mm·h^-1^ to 120 mm·h^-1^ in Xi et al. The rainfall in this study was natural, much less than its simulated rainfall, and its study also showed that the effect of cross ridge tillage on nitrogen loss was more pronounced under minor rainfall intensity conditions.

Compared with longitudinal ridge tillage, the concentration and amount of loss of AN, NN, and TN from bare land were slightly more significant than in longitudinal ridge tillage. Due to the longitudinal ridge tillage planting *Glycine max*, particulate nitrogen is intercepted by soybean stalks and fixed by soil adsorption [45]; likewise, *Glycine max* will have consumed some nutrients during the growth process. Due to the high vegetation cover, soil erosion in longitudinal ridge tillage is slightly better than bare ground, resulting in slightly lower nitrogen loss than bare ground [52]. Raindrops directly splash bare ground, and nutrients on soil particles and micro aggregates are broken down and absorbed, thus increasing the amount of nitrogen in the runoff [53].

The concentration and amount of TP lost by the extreme rainfall of 8–25 were much higher than other rainfall, and the loss of TP from bare land was severe (Fig 5); as phosphorus was primarily lost in the granular state, and the rainfall in this field caused more severe soil erosion [54]. Among them, the soil erosion of bare land is extremely serious, and its sediment production is twice as much as that of longitudinal ridge tillage and tens of times that of cross ridge tillage, leading to more severe TP loss. Fang [55] and Tang et al. [56] also considered bare land the central TP loss area. Compared with plot A_1_, the TP loss in plot A_2_ is reduced by 92.4~97.4%; compared with plot B_1_, the TP loss in cell B_2_ is reduced by 94.8%~98.7%. Since PP makes up a large percentage of TP and tends to adsorb to sediments, cross ridge tillage can reduce soil losses and thus significantly reduce TP losses. It can be seen that agricultural non-point source pollution is mostly produced by soil erosion as a carrier, and slowing down soil erosion can effectively prevent nitrogen and phosphorus loss.

### Percentage of loss of different forms of nitrogen and phosphorus

The loss of N is mainly in the dissolved state, i.e., the loss of N will respond more sensitively to the variations of runoff volume. The relationship between AN, NN, TN, and runoff was more significant, especially in the two bare land plots of A_3_ and B_3_ were highly significantly correlated, and the correlation coefficient was higher than 0.95. Nitrogen loss is mainly dominated by NN due to the negatively charged NN, which is easily soluble in water and constantly migrates with runoff [57], while the positively charged AN is more stable in combination with the soil and the more considerable loss occurs only under severe sediment erosion [58].

Due to the strong nitrification of AN under dry crop conditions, although the loss concentration and loss of AN and NN fluctuate with the variations of rainfall-runoff, on the contrary, the proportion of AN and NN loss to TN loss is more stable. After a long period of nitrification, the percentage of AN loss decreased from 10.9% ~ 14.9% to 6.3% ~ 8.1%, and the percentage of NN loss increased from 45.2% ~ 63.7% to 70.1% ~ 81.8%.

The loss flux of PP accounted for 72.7% to 96.2% of TP (Fig 6). PP and TP were highly correlated with SLR. The percentage of PP loss is more minor in cross ridge tillage and larger in bare land; compared with cross ridge tillage, bare land has a more significant flow-producing sediment production and severe soil erosion, and phosphorus bound to soil particles is easily lost with runoff [50]. The opposite result was obtained by He et al. [59], whose study showed that the loss flux of DTP was a more significant proportion of TP in surface runoff under cross ridge tillage in purple soil area. Cao et al. [60] explained the high rate of DTP output during the late rainfall period in their study, which concluded that the leaching force of runoff gradually increases with the increase of loamy midstream flow, thus leading to the phenomenon. In contrast, this study did not occur due to the fact that the rain intensity of natural rainfall was much less than that of simulated rainfall, followed by the thicker and more fertile soils in the black soil area.

## Conclusion

This study advances the study of agricultural nonpoint source pollution in black soil areas of China by exploring the soil erosion and nutrient loss characteristics of sloping cropland under different soil and water conservation measures. The RUSLE model has a better fit in the cross ridge tillage as well as in the bare ground. Nitrogen is mainly lost in the dissolved state with runoff. In contrast, phosphorus is easily adsorbed to sediments and thus lost with soil. The intensity of rainfall, slope, and vegetation cover significantly influence soil and nutrient loss. Changing longitudinal ridge tillage to cross ridge tillage is the most effective measure to prevent soil and nutrient loss, and this method has resulted in a reduced rate of over 90%. Planting plants with better water-holding and sediment-fixing properties, such as *Amorpha fruticosa Linn*., can achieve better results. Such measures are particularly effective in black soil areas with less rainfall intensity and slope gradient. Bare land and longitudinal ridge tillage land are still the focus of soil and nutrient loss control in black soil areas.

In future studies, the time span of the experiments needs to be increased and the parameters of the RUSLE model need to be determined more accurately. Similarly, different soil and water conservation measures need to be added to test the applicability of the RUSLE model in multiple situations. The ability of water retention and sediment fixation needs to be studied from different periods of crop growth. Soil and nutrient loss under different vegetation covers can also be explored. We also need to think about how to apply the data obtained from runoff plots to hydrological models.
